# Desensitization in patients with hypersensitivity to platinum and taxane in gynecological cancers

**DOI:** 10.1002/cam4.6840

**Published:** 2023-12-22

**Authors:** Tibor A. Zwimpfer, Kathrin Scherer, Andreas Schötzau, Viola Heinzelmann‐Schwarz, Karin Hartmann, Marcus Vetter, Céline Montavon

**Affiliations:** ^1^ Department of Gynecology and Gynecological Oncology, Hospital for Women University Hospital Basel Basel Switzerland; ^2^ Gynecological Cancer Center University Hospital Basel Basel Switzerland; ^3^ Peter MacCallum Cancer Center East Melbourne Victoria Australia; ^4^ Division of Allergy Unit, Department of Dermatology Cantonal Hospital Aarau Aarau Switzerland; ^5^ Department of Dermatology University Hospital Basel Basel Switzerland; ^6^ Cancer Center, Cantonal Hospital Baselland Medical University Clinic Liestal Switzerland

**Keywords:** chemotherapy, desensitization, gynecologic cancer, hypersensitivity reaction, platinum, taxane

## Abstract

**Background:**

Exposure to paclitaxel and carboplatin has the risk of developing hypersensitivity reactions (HSRs), which could necessitate using less effective treatments to avoid anaphylaxis. Desensitization to platinum and taxane HSRs can be used to complete chemotherapy according to the standard regimen; therefore, this study investigated rates and benefits of successful desensitization in patients with gynecologic cancers (GC).

**Methods:**

We collected data from 241 patients with GC who had at least one cycle of platinum or taxane chemotherapy. The rate of HSRs and successful desensitization were evaluated, and an outcome analysis was conducted.

**Results:**

The rate of HSRs to platinum and taxane was 6.39% and 13.07%, respectively. We observed a 100% success rate of desensitization in our cohort. Patients with HSR were significantly younger (57.1 vs. 64.9 years, *p* = 0.030) in the taxane cohort. Importantly, the overall survival (OS) of patients with platinum and taxane HSRs who underwent desensitization was comparable to that of patients with no HSRs (platinum vs. controls; median OS 60.36 vs. 60.39 months, *p* = 0.31; taxane vs. controls; OS 80.29 vs. 60.00 months, *p* = 0.59).

**Conclusion:**

Thus, we show that desensitization for platinum and taxane HSRs is safe and effective, resulting in an outcome that is well comparable to patients without HSR. Based on these observations, desensitization procedures might be considered as standard of care before switching to less effective treatment for patients with GC.

## 1 INTRODUCTION

Platinum‐ and taxane‐based chemotherapy is standard of care in patients with advanced gynecologic cancers (GC) including epithelial ovarian, tubal and peritoneal cancers (EOCs), advanced/metastatic endometrial cancer (EC), and cervical cancer (CC).^1^, ^2^, ^3^, ^4^ Based on current guidelines, patients with a relapse of their disease are rechallenged with platinum‐based chemotherapy as long as no resistance has developed defined by clinical, biochemical, and radiologic examinations.^1^ Moreover, for patients with platinum‐resistant ovarian cancer, paclitaxel is the most commonly used drug.^5^

Today, more treatment lines are applied in GC than 1–2 decades before.^6^ Multiple exposures to the same agent, such as platinum and taxane, induce oncological resistance with an increase of antioxidant response of the cancer cells^7^, ^8^ and can also result in allergic hypersensitivity reactions (HSRs), which affect further treatment and outcomes by necessitating a switch to a less effective and more toxic chemotherapy regimen.^9^, ^10^ The antineoplastic agents platinum and taxane together with L‐asparaginase and epipodophyllotoxins have the highest frequency of HSRs.^11^, ^12^, ^13^ Approximately 5% of the general oncologic population and 8%–16% of the GC patients are affected by platinum hypersensitivity and 10% in both populations experience taxane hypersensitivity.^14^, ^15^, ^16^, ^17^ This is clinically meaningful and a strategy to maintain the optimal treatment regimen is warranted.

In cases of mild HSRs, premedication with antihistamines and corticosteroids is typically recommended and, in the case of taxanes and platinum, routinely performed.^11^, ^12^, ^13^, ^18^ However, premedication is ineffective in preventing more severe allergic reactions, especially reactions to platinum salts.^19^, ^20^

Desensitization, or synonymous tolerance induction, is a procedure for establishing a temporary tolerance to a substance that has triggered an HSR in the past.^21^ It should be considered in patients with HSRs to platinum salts and taxanes as it is a safe alternative when conducting standard chemotherapy that aims for the best therapeutic result according to international standards.^14^, ^15^, ^22^, ^23^ Currently, knowledge concerning desensitization procedures is established and international guidelines for their management exists.^24^ Furthermore, an improved outcome for overall survival (OS) has been demonstrated in hypersensitive patients receiving carboplatin desensitization compared to non‐hypersensitive patients in recurrent ovarian cancer, independent of germline *BRCA* status.^25^ However, there is a scarcity of specialized centers that offer desensitization as part of the standard procedure and investigations into the clinical effect of desensitization is uncommon. It is important to analyze and optimize desensitization protocols and to test and ensure their safety and efficacy, including survival data.

The objectives of this study were to (1) determine the prevalence of HSRs to platinum and/or taxane chemotherapy in patients with GC, (2) analyze the rate and outcome of successful desensitization, and (3) compare the clinical outcome of patients with HSRs to patients without reactions in terms of recurrence‐free survival (RFS) and OS.

## 2 METHODS AND MATERIALS

### 2.1 Study design

We conducted a retrospective, single‐center cohort study from 2012 to 2021 at the Department of Gynecologic Oncology, University Hospital Basel. We identified and compared patients with HSRs to platinum and/or taxane chemotherapy to patients without reactions and analyzed the rate and outcome of successful desensitization and the clinical outcome in terms of OS.

### 2.2 Study population and setting

We analyzed the clinicopathological data of 241 patients older than 18 years with GC (including epithelial ovarian cancer, peritoneal cancer, fallopian tube cancer, advanced/recurrent endometrial cancer, and advanced/recurrent cervical cancer) treated with at least one cycle of platinum‐ and/or taxane ‐based chemotherapy between January 2012 and December 2019 at the Department of Gynecologic Oncology, University Hospital Basel. Carboplatin and paclitaxel were given to all patients receiving platinum‐ and taxane‐based chemotherapy, respectively. In addition, for one line of the total regimen, 2 patients received nanoparticle albumin‐bound paclitaxel (nab‐paclitaxel) and 29 patients received cisplatin. Ethics approval was obtained from the Ethical Committee of Nordwest‐ und Zentralschweiz, Switzerland (EKNZ 2020‐00160). All patients signed a general consent form, which included further use of health‐related data. The anonymization of personal data was guaranteed. The whole study was performed according to the Declaration of Helsinki as well as local laws and regulations.

### 2.3 Variables

GC included epithelial ovarian, peritoneal, fallopian tube, endometrial, and cervical cancers. Based on the received chemotherapy, we divided the patients into platinum (*n* = 219) and taxane (*n* = 153) groups and compared the rate of HSRs, desensitization, and outcomes for all gynecological cancers. Furthermore, we performed a subgroup analysis for the EOC patients. We did not perform a subgroup analysis for cervical and endometrial cancer due to the small sample size and associated low statistical power.

### 2.4 Data sources/measurement

Patient's data were collated from the hospital's clinical portal record. Information collected included patient demographic data, disease characteristics, and follow‐up data until data cutoff in November 2021.

All patient's data related to their personal and medical history, as well as the operative and oncological therapy and follow‐up were documented in an electronic patient chart. Detailed information on chemotherapeutic treatment (regimen, date, dose, etc) were recorded within the hospital in an electronic secured system named “CATO,” used to order chemotherapy in the hospital pharmacy. All data related to their allergy workup was documented in paper charts and—in part—archived as pdf‐scans in the electronic chart. These data were collected and shared by the gynecological and medical oncologist, allergist, and pharmacist.

All patients who developed an HSR to any platinum salt or taxane were referred to our Division of Allergy within 1–2 weeks following the reaction and were examined by a specialized allergologist. In addition to a thorough medical history with a special emphasis on allergic diseases of any kind, symptoms were assessed in detail and the reactions were classified in accordance with Ring and Messmer (Table S1).^26^ Potential cofactors (e.g., infection and nonsteroidal anti‐inflammatory drugs), aggravating factors (simultaneous intake of angiotensin converting enzyme‐inhibitors or beta blockers, stress, mast cell disease) were assessed as well as concomitant diseases (e.g., asthma and atopy). Patients were then skin‐tested with the aim to establish the putative immunologic mechanism of the reaction. Skin prick tests and intradermal tests were performed as recommended in the literature using histamine as a positive and saline solution as a negative control.^27^ Baseline mast cell tryptase was measured in most cases. Depending on the results of the skin tests and the severity of the initial clinical reaction, patients were scheduled for a tolerance induction/desensitization procedure with the chemotherapeutic agent (platinum or taxane), either in the gynecological oncology outpatient clinic (mild initial reaction, skin test negative, and no potential cofactors) or in the allergy clinic (severe initial reaction, skin test positive, and potential cofactors) as outlined in Figure S1.

Desensitization protocols for immediate type HSRs to chemotherapeutic agents were based on the stepwise increase of infusion rates of highly diluted drug solutions, starting as slowly as only a few microgram per millilitre of the drug in the first hour. The desensitization protocols were part of 12‐step and 16‐step protocols as previously described.^14^, ^21^, ^28^ If an initial administration of medication was well tolerated, a slightly shorter protocol (8–10 steps) was attempted (Figure S1 and Supplementary Material 1).

The choice of protocol for a particular desensitization was based on risk stratification from the skin test results, the severity of the initial clinical reaction, tryptase levels, and potential underlying diseases, which could have affected the risk of renewed allergic reactions.

Successful desensitization was defined as completed desensitization protocol to platinum and/or taxane and completed chemotherapy according to the standard regimen.

### 2.5 Statistical analyses

Descriptive statistics are presented as counts and frequencies for categorical data and medians (range) for metric or ordinal variables. In case of medians *p*‐values correspond to the Kruskall–Wallis tests, in case of categorical data *p*‐values correspond to Fisher's exact tests. For RFS and OS, Kaplan–Meier estimates were calculated for each HSR group, with estimated times at RFS or OS probabilities of 0.5 (median) and 0.75 (75 quantile). *p*‐values of group comparisons correspond to log‐rank tests. A *p‐*value <0.05 was considered significant. All analyses were performed using the statistical software R version 4.1.3.

## 3 RESULTS

### 3.1 Platinum group

The platinum group (all patients received carboplatin, dosed by area under the curve (AUC)) consisted of 126 patients (57.5%) with EOC, 48 patients with EC (21.9%), and 45 patients with CC (20.5%). Demographic and clinicopathological baseline characteristics for these patients are shown in Table 1 and were well balanced except of a trend to younger age in the patients with HSR (*p* = 0.065).

**TABLE 1:** Comparison of the demographic and clinicopathologic characteristics between patients with platinum‐related HSRs and those with no HSRs in gynecologic cancers (epithelial ovarian/peritoneal/fallopian tube, endometrial, and cervical).

Characteristics	All patients (*n* = 219) *n* (%)	No HSR (*n* = 205) *n* (%)	HSR to platinum (*n* = 14) *n* (%)	*p*‐value^a^
Age at diagnosis (years)				
Median	63.7	64.4	56.3	0.065
Range	29.1–88.4	29.1–88.4	37.1–84.6	
Primary site				
Ovary/peritoneum/fallopian tube	126 (57.5)	117 (57.1)	9 (64.3)	0.056
Corpus uteri	48 (21.9)	48 (23.4)	0 (0.00)	
Cervix uteri	45 (20.5)	40 (19.5)	5 (35.7)	
FIGO stage EOC				
I + II	29 (23.0)	1 (11.1)	28 (23.9)	0.683
III + IV	97 (77.0)	8 (88.9)	89 (76.1)	
Grade EOC				
1	7 (5.02)	7 (5.98)	0 (0)	1.000
2	3 (14.6)	3 (2.56)	0 (0)	
3	115 (77.6)	106 (90.6)	9 (100)	
Unknown	1 (2.74)	1 (0.85)	0 (0)	
FIGO stage EC				
I + II	20 (41.7)	20 (41.7)	0 (0)	–
III + IV	28 (58.3)	28 (58.3)	0 (0)	
Grade EC				
1	2 (4.17)	2 (4.17)	0 (0)	–
2	12 (25.0)	12 (25.0)	0 (0)	
3	33 (68.8)	33 (68.8)	0 (0)	
Unknown	1 (2.08)	1 (2.08)	0 (0)	
FIGO stage CC				
I + II	24 (53.3)	22 (55.0)	2 (40.0)	0.652
III + IV	21 (46.7)	18 (45.0)	3 (60.0)	
Grade CC				
1	2 (4.44)	2 (5.0)	0 (0)	0.495
2	17 (37.8)	16 (40.0)	1 (20.0)	
3	22 (48.9)	19 (47.5)	3 (30.0)	
Unknown	3 (8.89)	2 (7.5)	2 (20.0)	
Ethnicity				
Caucasian	198 (92.5)	184 (92.0)	14 (100)	1.000
Hispanic	7 (3.27)	7 (3.50)	0 (0.00)	
Asian	9 (4.21)	9 (4.50)	0 (0.00)	
Family history of gynecologic cancer	74 (35.1)	70 (35.5)	4 (28.6)	0.775
Concurrent taxane chemotherapy	144 (73.1)	131 (71.6)	13 (92.9)	0.118
Lines of chemotherapy				
Median	1	1	3.5	**<0.001**
Range	1–7	1–7	1–7	
Cycles of chemotherapy				
Median	6	6	11.5	**0.001**
Range	1–24	1–24	2–20	
Cycles of chemotherapy until HSR				
Median	–	–	8	–
Range	–	–	4–11	
Cumulative dose of platinum (mg)				
Median	3140	3066	4261	0.092
Range	180–13,470	180–13,470	466–11,130	

Abbreviations: CC, cervical cancer; EC, endometrial cancer; EOC, epithelial ovarian cancer; HSR, hypersensitivity reaction; mg, milligram; *n*, number of patients.

^a^ The *p*‐values were calculated using Kruskal–Wallis test (medians) or Fisher's exact test (categorical data). A *p*‐value <0.05 was considered significant.

#### 3.1.1 HSR and desensitization

**FIGURE 1:**
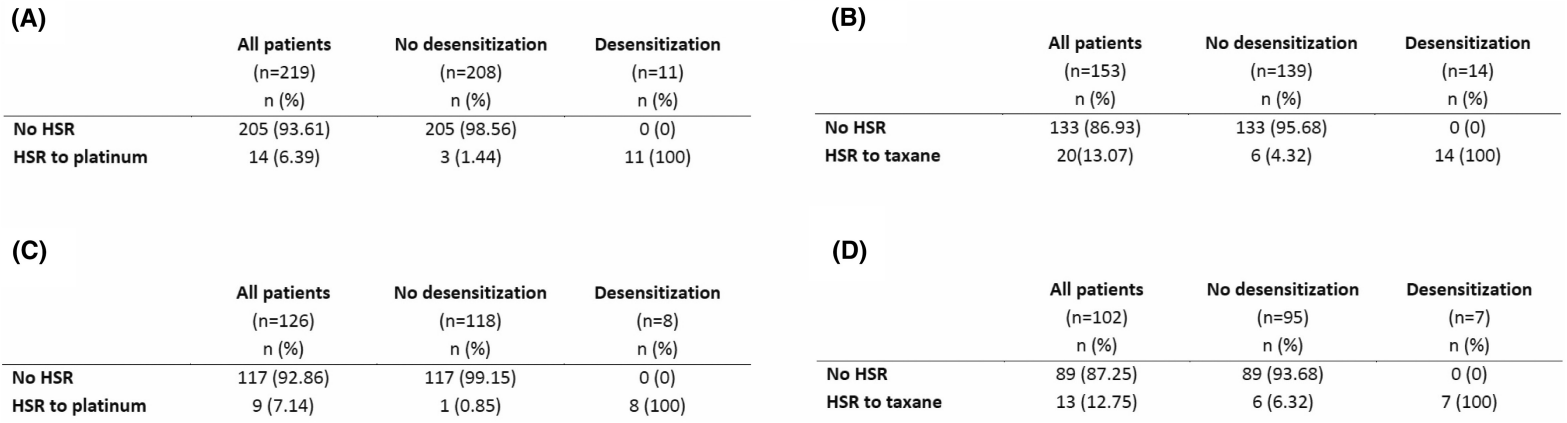
Rates of HSR and desensitization to (A) platinum‐ and (B) taxane‐based chemotherapy in GC (epithelial ovarian/peritoneal/fallopian tube, endometrial, and cervical) and (C) to platinum‐ and (D) taxane‐based chemotherapy in ovarian cancer. HSR, hypersensitivity reaction; *n*, number of patients.

Out of 219 patients receiving platinum‐based chemotherapy, 14 (6.39%) had an HSR to carboplatin (Figure 1). Grade 2 was the most frequent level of allergy with 71.4% and the patients experienced most commonly respiratory (71.4%) and skin symptoms (71.4%) (Table 2). HSRs to platinum occurred after a median of eight cycles of platinum‐based chemotherapy (Table 1).

**TABLE 2:** Severity and symptoms of HSRs in patients with HSRs to platinum and taxane.

Parameter	All patients (*n* = 34) *n* (%)	Platinum (*n* = 14) *n* (%)	Taxane (*n* = 20) *n* (%)	*p*‐value^a^
Grade of HSRs				
Grade 1	5 (14.7)	1 (7.14)	4 (20)	0.336
Grade 2	18 (52.9)	10 (71.4)	8 (40)	
Grade 3	6 (17.6)	1 (7.14)	5 (25)	
Grade 4	1 (2.94)	0 (0)	1 (5)	
Unknown	4 (11.8)	2 (14.3)	2 (10)	
Symptoms				
Skin	16 (47.1)	10 (71.4)	6 (30)	0.066
Respiratory	19 (55.9)	10 (71.4)	9 (45)	0.173
Gastrointestinal tract	9 (26.5)	4 (28.6)	5 (25)	0.677
Cardiovascular	8 (23.5)	2 (14.3)	6 (30)	0.277
Others	19 (55.9)	7 (50)	12 (60)	0.715

Abbreviations: HSR, hypersensitivity reaction; *n*, number of patients.

^a^ *p*‐values were calculated using Fisher's exact test. A *p*‐value <0.05 was considered significant.

Desensitization was conducted in 11 out of 14 patients (78.57%) with HSRs to platinum with a success rate of 100%, meaning that all patients treated could accomplish their standard of care. However, 4 out of these 11 patients (36.4%) had a breakthrough reaction that required specific management in our Division of Allergy. There was no statistically significant difference in OS (*p* = 0.26) between the patients with breakthrough vs no breakthrough reaction. Among the 14 patients with an HSR to platinum, 3 patients (21.43%) had no desensitization. Given the mild nature of the symptoms, all three patients continued the chemotherapy without desensitization regime.

There was a trend for patients with HSRs being younger compared to those with no HSRs in the platinum cohort (56.3 vs. 64.4 years, *p* = 0.065). Additionally, patients with HSRs received significantly more lines of chemotherapy with a median of 3.5 lines compared to 1 line in patients without HSRs (*p* < 0.001). Therefore, patients with HSRs had significantly more cycles and a higher cumulative dose of platinum compared to patients with no HSRs, (*p* < 0.001 and *p* = 0.092, respectively) as shown in Table 1.

#### 3.1.2 Overall survival and recurrence‐free survival

**FIGURE 2:**
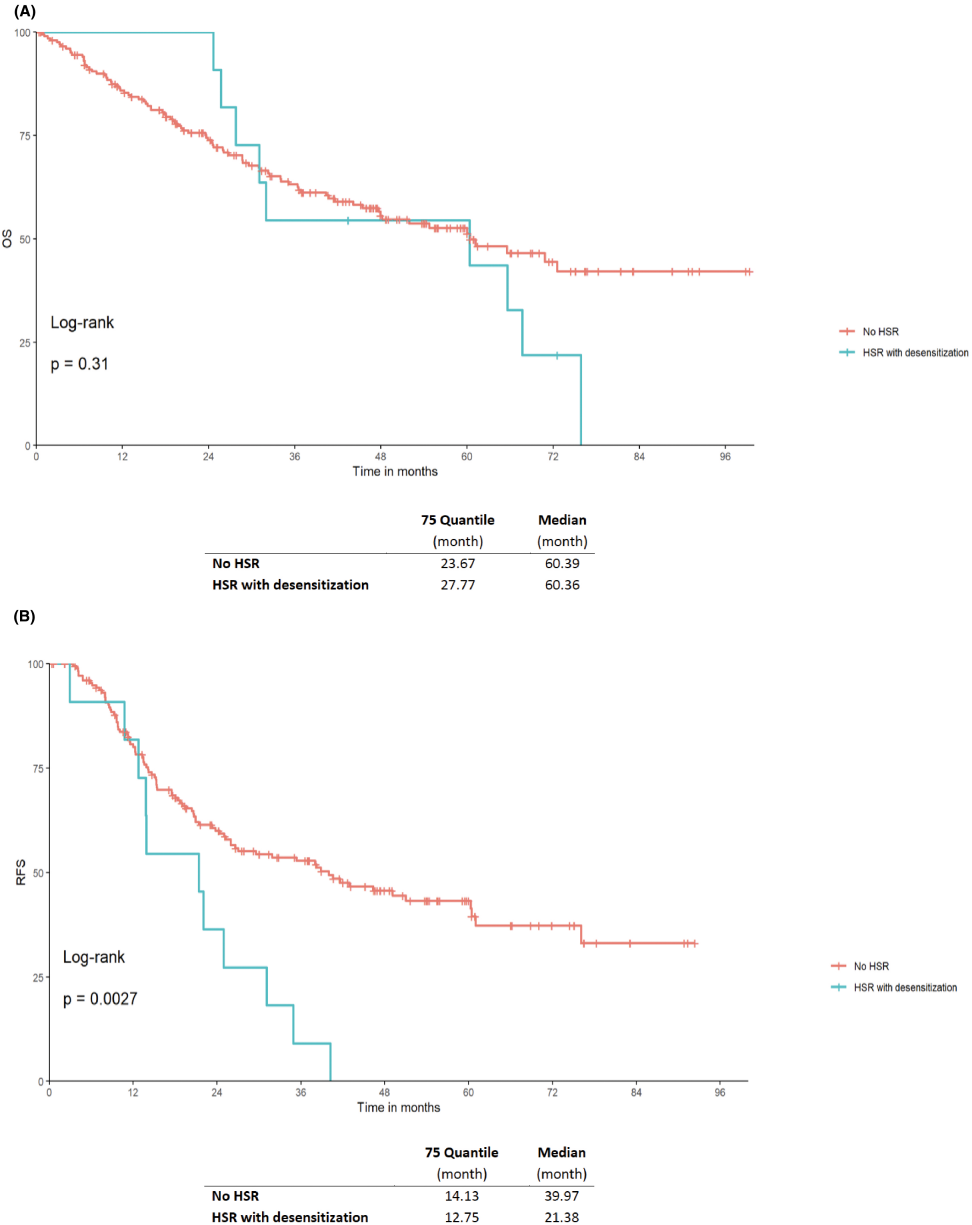
Kaplan–Meier curves of (A) OS and (B) RFS for patients with platinum‐based chemotherapy and no hypersensitivity reaction (HSR) compared to patients with an HSR and successful desensitization and continuation of platinum chemotherapy for gynecologic cancers. A *p*‐value <0.05 was considered significant. HSR, hypersensitivity reaction; OS, overall survival; RFS, recurrence‐free survival.

There was no significant difference in OS (*p =* 0.31) in patients with no HSRs (median of 60.39 months) compared to patients with HSRs to platinum and desensitization (median of 60.36 months). However, RFS in patients with no HSRs was significantly longer *(p =* 0.0027) with a median of 39.97 months compared to patients with HSRs to platinum and desensitization (median of 21.38 months) (Figure 2).

### 3.2 Taxane group

The taxane group (all patients received paclitaxel) consisted of 102 patients (66.7%) with EOC, 35 patients with EC (22.9%), and 16 patients with CC (10.5%). Demographic and clinicopathological baseline characteristics for these patients are reported in Table 3. The mean age was 63.0 years. Of these patients, 99.3% also had concomitant platinum‐based chemotherapy.

**TABLE 3:** Comparison of the demographic and clinicopathologic characteristics between patients with taxane‐related HSRs and those with no HSRs in gynecologic cancers (epithelial ovarian/peritoneal/fallopian tube, endometrial, and cervical).

Characteristics	All patients (*n* = 153) *n* (%)	No HSR (*n* = 133) *n* (%)	HSR to taxane (*n* = 20) *n* (%)	*p*‐value^a^
Age at diagnosis (years)				
Median	63.0	64.9	57.1	**0.030**
Range	30.5–85.5	30.5–85.5	40.2–71	
Primary site				
Ovary/peritoneum/fallopian tube	102 (66.7)	89 (66.9)	13 (65)	0.056
Corpus uteri	35 (22.9)	30 (22.6)	5 (25)	
Cervix uteri	16 (10.5)	14 (10.5)	2 (10)	
FIGO stage EOC				
I + II	23 (22.5)	17 (19.1)	6 (46.2)	0.068
III + IV	79 (77.5)	72 (80.9)	7 (53.8)	
Grade EOC				
1	5 (4.9)	5 (5.62)	0 (0)	0.126
2	2 (1.96)	1 (1.12)	1 (7.69)	
3	93 (91.2)	82 (92.1)	11 (84.6)	
Unknown	2 (1.96)	1 (1.12)	1 (7.69)	
FIGO stage EC				
I + II	8 (22.9)	8 (26.7)	0 (0)	0.315
III + IV	27 (77.1)	22 (73.3)	5 (100)	
Grade EC				
1	2 (5.71)	2 (6.67)	0 (0)	1.000
2	9 (25.7)	8 (26.7)	1 (20)	
3	24 (68.6)	20 (66.7)	4 (80)	
FIGO stage CC				
I + II	5 (31.2)	5 (35.7)	0 (0)	1.000
III + IV	11 (68.8)	9 (64.3)	2 (100)	
Grade CC				
1	1 (6.25)	1 (7.14)	0 (0)	0.625
2	5 (31.2)	5 (35.7)	0 (0)	
3	9 (56.2)	7 (50)	2 (100)	
Unknown	1 (6.25)	1 (7.14)	0 (0)	
Ethnicity				
Caucasian	136 (90.1)	119 (90.2)	17 (89.5)	0.472
Hispanic	8 (5.3)	6 (4.55)	2 (10.5)	
Asian	6 (3.97)	6 (4.55)	0 (0.00)	
Black‐African	1 (0.66)	1 (0.76)	0 (0.00)	
Family history of gynecologic cancer	52 (35.4)	47 (36.2)	5 (29.4)	0.782
Concurrent platinum chemotherapy	151 (99.3)	131 (99.2)	20 (100)	0.118
Lines of chemotherapy				
Median	1	1	1	0.552
Range	1–7	1–7	1–4	
Cycles of chemotherapy				
Median	6	6	6	0.164
Range	1–13	1–13	1–12	
Cycles of chemotherapy until HSR				
Median	–	–	1.5	–
Range	–	–	1–3	
Cumulative dose of platinum (mg)				
Median	1745	1745	1715	0.548
Range	1–8360	1–8360	280–2920	

Abbreviations: CC, cervical cancer; EC, endometrial cancer; EOC, epithelial ovarian cancer; HSR, hypersensitivity reaction; mg, milligram; *n*, number of patients.

^a^ The *p*‐values were calculated using Kruskal–Wallis test(medians) or Fisher's exact test(categorical data). A *p*‐value <0.05 was considered significant.

#### 3.2.1 HSR and desensitization

Overall, 153 patients received taxane‐based chemotherapy and 20 patients (13.07%) had an HSR to paclitaxel with a peak incidence after a median of 1.5 cycles of taxane‐based chemotherapy. The most common severity grade and symptom of the HSR were grade 2 (40%) and respiratory (45%), respectively (Table 2). Fourteen patients with an HSR to taxane (70%) went through desensitization, of which all were successful with no breakthrough reactions. The patients received a median of 1262 milligram (mg) and four cycles of taxane‐based chemotherapy after desensitization. Six patients (30%) with an HSR had no desensitization. Of these 6 patients, three patients preferred to change therapy to nab‐paclitaxel due to convenience and fear of a new reaction and the other three continued the chemotherapy due to mild symptoms and after intensifying the premedication. However, patients with HSRs were significantly younger compared to those without HSRs (*p* = 0.030). No other risk factors were identified as shown in Table 3.

#### 3.2.2 Overall survival and recurrence‐free survival

**FIGURE 3:**
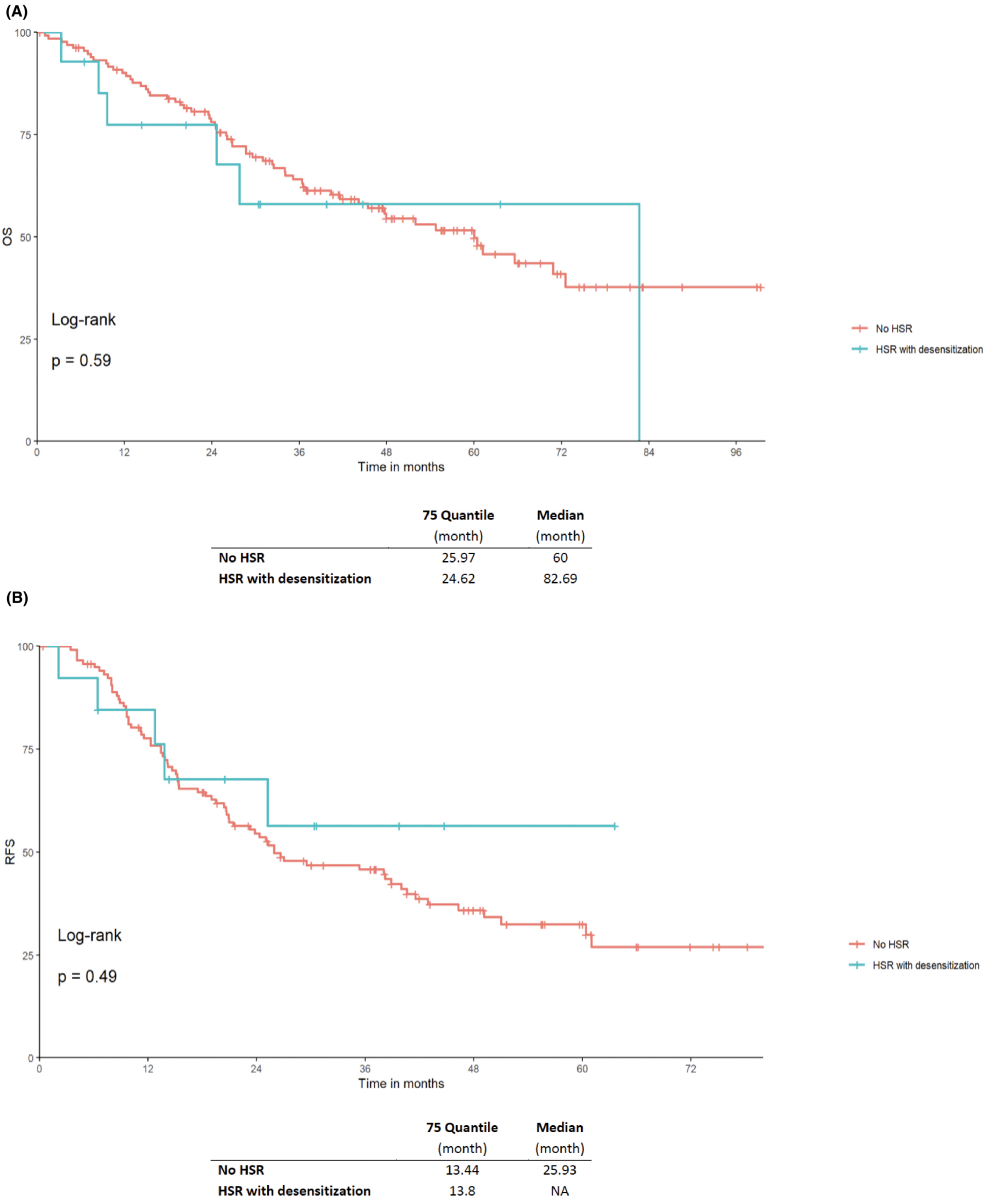
Kaplan–Meier curves of (A) OS and (B) RFS of patients with taxane‐based chemotherapy and no hypersensitivity reaction (HSR) compared to patients with HSRs and successful desensitization and continuation of taxane chemotherapy in gynecologic cancers. A *p*‐value <0.05 was considered significant. HSR, hypersensitivity reaction; OS, overall survival; RFS, recurrence‐free survival.

There were no significant differences in OS (*p =* 0.59) and RFS (*p =* 0.49) in patients without HSRs compared to those with HSRs to taxane and desensitization with a median OS of 60 versus 82.29 months, respectively (Figure 3).

### 3.3 Ovarian cancer patients

In total, 126 EOC patients with platinum‐based chemotherapy with an HSR rate of 7.14% (*n* = 9) and 102 EOC patients with taxane‐based chemotherapy with an HSR rate of 12.7% (*n* = 13) were included. Desensitization was performed in eight patients (88.9%) with HSRs to platinum and seven patients (53.89%) with HSRs to taxane. The rate of successful desensitization was 100%. There was no correlation found between HSR and BRCA, residual disease, and advanced stage. Patients with HSRs were significantly younger compared to those with no HSRs in the taxane cohort (*p* = 0.032) as shown in Table S2. No further risk factor was identified. Compared to patients with HSRs, patients without HSRs showed a significant difference in received platinum‐based chemotherapy lines (*p <* 0.001), cycles (*p <* 0.002), and cumulative dose *(p =* 0.019) in the platinum cohort, whereas in the taxane cohort there was no significant difference as shown in Tables S2 and S3.

There was no significant difference in OS between patients with and without HSRs to platinum and desensitization, or taxane and desensitization. However, RFS in patients with no HSRs was significantly longer (*p =* 0.042) compared to those with HSRs to platinum and desensitization (Figures S2 and S3).

## 4 DISCUSSION

Our data show that desensitization to platinum and taxane in GC patients with HSRs is safe, feasible, and yields a comparable OS in hypersensitive patients receiving continuous platinum and taxane chemotherapy to patients without HSRs. Patients with HSRs to platinum represent a group with a significant higher risk for recurrence, as the HSR to platinum occurs after a median amount of eight cycles of chemotherapy, making recurrence almost a conditio sine qua non for platinum HSR. However, the patients with platinum HSR and desensitization received a higher total dose of carboplatin compared to the nonreactive population and a higher number of median lines, again showing that this group recurred more often, but also remained carboplatin‐sensitive and therefore benefited the most from this crucial agent. This is particularly true for the ovarian cancer subgroup, and stresses the importance of desensitization in this population, due to the fact that response to platinum is the major prognostic factor for long‐term outcomes in EOC.^29^

The rate of HSRs to platinum‐and taxane‐based chemotherapy (6.39% and 13.07%, respectively) in our cohort correlates with the previously reported incidence for GC (8%–16% and 10%, respectively).^12^, ^13^, ^14^, ^15^ Incidence rates may be affected by premedication with steroids and antihistamines and slower administration rates of chemotherapeutic agents.^30^, ^31^ Hence, real HSR rates are likely to be underestimated, as oncologists often report only severe reactions.^19^, ^20^

HSRs to platinum occurs most frequently at the beginning of the second line of treatment, with a peak incidence at the eighth cycle, which correlates with data indicating that reexposure to platinum is associated with a high rate of HSRs.^32^ In our platinum group, patients with HSR have more recurrences and received a median of 3.5 lines and 11.5 cycles of platinum‐based chemotherapy compared to patients without HSR with a median of 1 line and 6 cycles, respectively (Table 1). This might explain the significantly longer RFS (*p* = 0.0027) in patients without HSR, but without impact on the OS (*p* = 0.31) (Figure 2). In contrast, HSRs to taxane showed a peak incidence at the second cycle, as also previously reported with no significant difference in OS (*p =* 0.59) and RFS (*p =* 0.49).^31^ The onset of HSRs to platinum and taxane could well be a result of the different mechanisms of hypersensitivity. HSRs to platinum is primarily immunglobulin E (IgE)‐mediated, especially in the more severe cases, and may direct mast cell activation.^14^, ^15^ IgE‐mediated reactions never occur upon first contact, thus, there is a clinically silent sensitization phase required for this immunological mechanism. HSRs to taxane is rather provoked by direct mast cell and complement activation,^33^ and only in some cases, specific IgE‐mediated mechanisms are involved.^34^ The detailed mechanisms underlying hypersensitivity to taxanes remains to be established.^31^

In our cohort, the age appears to be an independent risk factor for HSRs to taxane and a trend was observed in patients with HSRs to platinum. The previously reported correlation with BRCA mutation as risk factor for platinum or taxane HSR was not observed here and no additional risk factor was identified.^12^, ^25^, ^35^

We provide a comprehensive overview of HSR and desensitization according to current protocols in the most common GC with an in‐depth survival analysis. Additionally, we achieved an impressive success rate in our cohort, so that all patients with HSR and desensitization finished their treatment according to the standard of care without severe incidents for platinum and no incidents for taxane desensitization.

The small cohort size limits the statistical power of the results. For example, the small number of events may have affected the power of the study and hence, the results. This is partly the result of the prevalence of HSRs to platinum and taxane and partly to the strict exclusion criteria for patients with no signed general consent form. Another limitation concerns the inherent bias of retrospectively analyzed data. Thus, a long study period, incomplete data, potential referral bias, heterogeneous therapies, varying follow‐up practice patterns, and unidentifiable biases may exist. The prevention of these biases can only be accomplished with a prospectively randomized study.

Currently, desensitization protocols for patients with taxane‐and platinum HSRs are available and recommended,^11^, ^12^, ^13^, ^24^ but a limited number of cancer centers have established desensitization as part of their standard procedures. The analysis and management of successful tolerance induction in patients with HSRs to carboplatin and taxane should be regularly applied in the medical setting, and the knowledge of desensitization procedures in gynecologic oncology could be optimized. This is important for achieving optimal treatment according to international standards.^19^, ^20^ However, it is crucial not to delay planned chemotherapy for desensitization as the goal is to provide the best treatment within the recommended time schedule. Therefore, patients developing an HSR should be seen and tested by an allergist within 1–2 weeks after a reaction. If there is a contrary indication to desensitization, there is still the option to change the therapy to a similar chemotherapy, for instance to oxaliplatin or nab‐paclitaxel. With all the new agents available in the future, including immune checkpoint inhibitors, antibody‐drug conjugates, anti‐angiogenetic agents, PARP‐inhibitors and small molecules, the rechallenge rate may decrease, but it will still be important to have the option of rechallenge with platinum‐ and taxane‐based therapies.

The outcome analysis is of importance, particularly as there are only few data reporting on the oncological impacts of the desensitization procedure in HSRs to either platinum or taxane. Our data suggest that HSR is not detrimental to oncological outcome in patient with GC, and more particularly with ovarian cancer, when a desensitization procedure is performed according to a rigorous protocol in a specialized or trained unit. Therefore, our study emphasizes the clinical importance of recruiting patients with HSRs for desensitization as opposed to switching to alternative therapies.

## AUTHOR CONTRIBUTIONS

**Tibor Zwimpfer:** Data curation (equal); formal analysis (lead); funding acquisition (equal); investigation (equal); methodology (equal); visualization (equal); writing – original draft (equal); writing – review and editing (equal). **Kathrin Scherer:** Conceptualization (equal); methodology (supporting); resources (equal); writing – original draft (equal); writing – review and editing (equal). **Andreas Schötzau:** Formal analysis (equal); methodology (equal); software (lead); visualization (lead); writing – original draft (equal); writing – review and editing (equal). **Viola Heinzelmann‐Schwarz:** Conceptualization (equal); methodology (equal); project administration (equal); resources (equal); supervision (equal); writing – original draft (equal); writing – review and editing (equal). **Karin Hartmann:** Conceptualization (equal); investigation (equal); project administration (equal); writing – original draft (equal); writing – review and editing (equal). **Marcus Vetter:** Conceptualization (equal); formal analysis (equal); project administration (equal); supervision (equal); writing – original draft (equal); writing – review and editing (equal). **Céline Montavon:** Conceptualization (lead); data curation (supporting); formal analysis (supporting); investigation (supporting); methodology (equal); project administration (lead); resources (equal); supervision (lead); writing – original draft (equal); writing – review and editing (equal).

## FUNDING INFORMATION

This work was supported by the Freie Gesellschaft Basel Gottfried und Julia Bangerter‐Rhyner‐Stiftung 0297 Schweizerischer Nationalfonds zur Förderung der Wissenschaftlichen Forschung P500PM_20726.

## CONFLICT OF INTEREST STATEMENT

The authors declare that they have no conflicts of interest or financial ties to disclose.

## ETHICS APPROVAL AND CONSENT TO PARTICIPATE

Ethics approval was obtained from the Ethical Committee of Nordwest‐ und Zentralschweiz, Switzerland (EKNZ 2020–00160). All participants gave their written consent to participate in this study and waived any claims. The anonymization of personal data was guaranteed.

## Supporting information


Figure S1.

Figure S2.

Figure S3.

Table S1.

Table S2.

Table S3.

Data S1.

Figure Captions.

## Data Availability

The datasets that have been used and/or analyzed during the current study are available from the corresponding author on reasonable request.
