# Supplementary figures and images for: Desensitization in patients with hypersensitivity to platinum and taxane in gynecological cancers
Source: Cancer Med. 2023 Dec 22;13(1):e6840. doi: 10.1002/cam4.6840 (PMC10807606; doi:10.1002/cam4.6840)

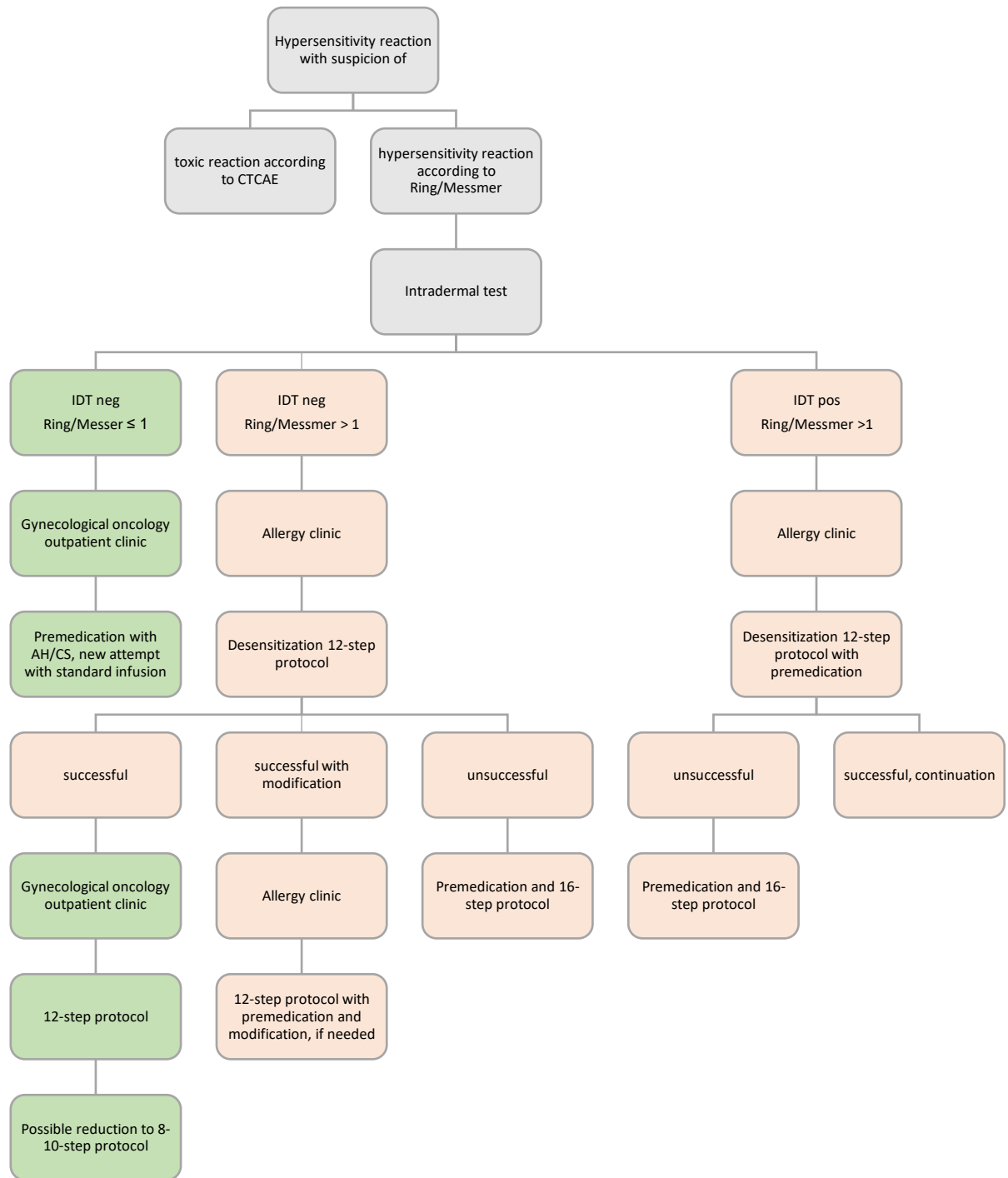

Supplement: Supplementary file 1 — Figure S1. [file CAM4-13-e6840-s003.pdf]

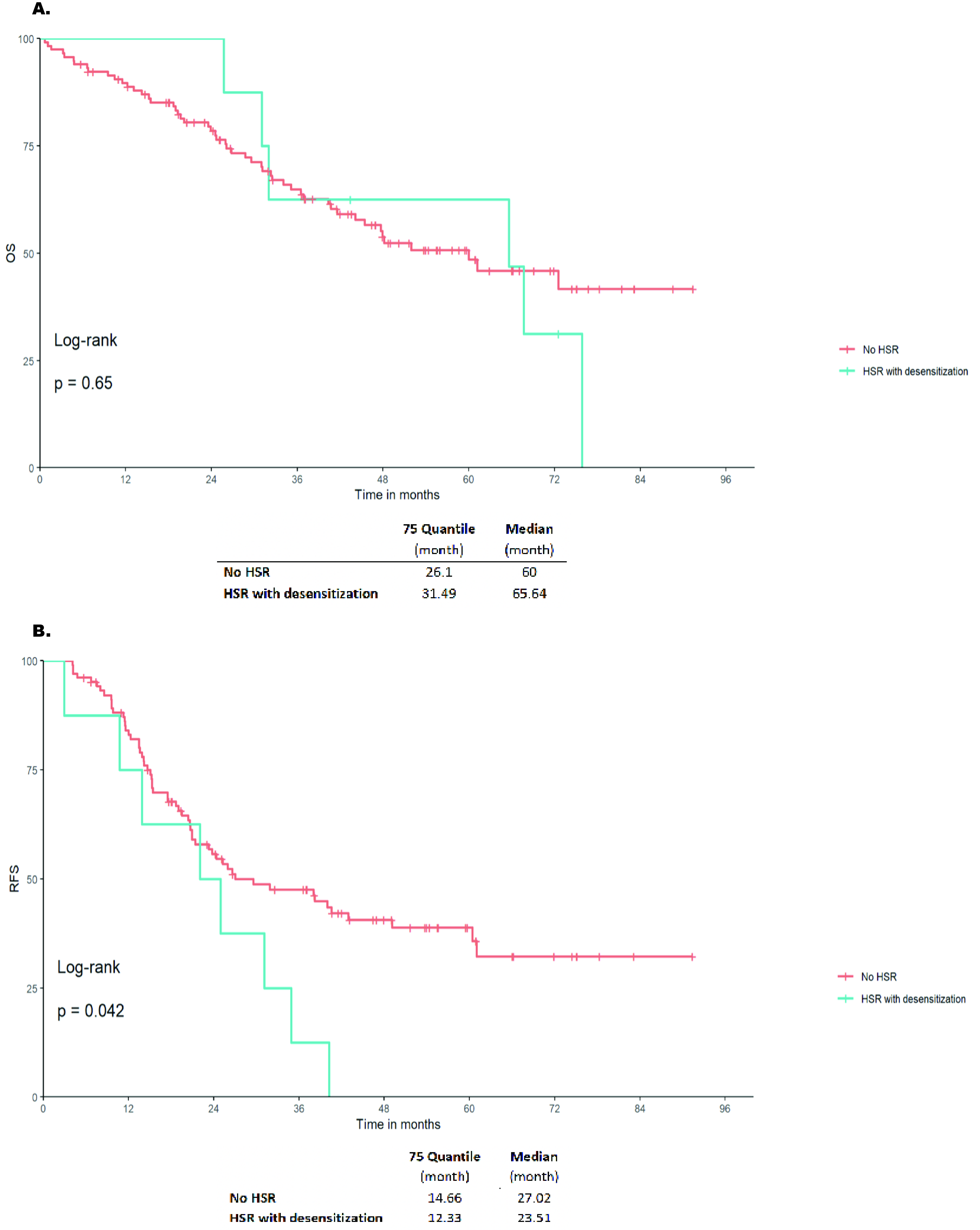

Supplement: Supplementary file 2 — Figure S2. [file CAM4-13-e6840-s008.jpg]

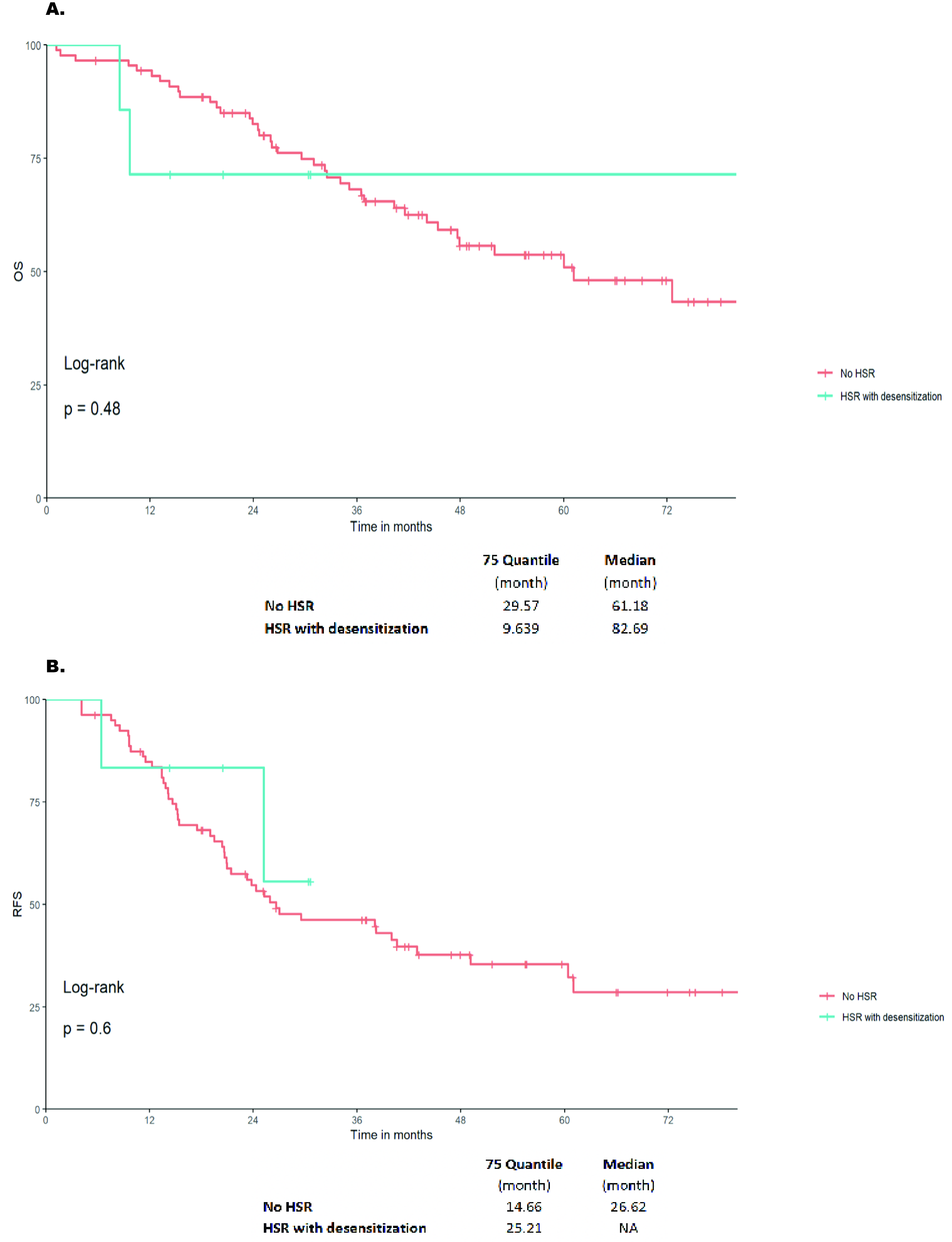

Supplement: Supplementary file 3 — Figure S3. [file CAM4-13-e6840-s002.jpg]
