# Supplementary material for: Desensitization in patients with hypersensitivity to platinum and taxane in gynecological cancers
Source: Cancer Med. 2023 Dec 22;13(1):e6840. doi: 10.1002/cam4.6840 (PMC10807606; doi:10.1002/cam4.6840)
Supplement: Supplementary file 4 — Table S1. [file CAM4-13-e6840-s007.docx]

**Table S1.** Immediate type of hypersensitivity reaction - classification according to Ring and Messmer.

| **Grade** | **Skin** | **Abdomen** | **Respiratory tract** | **Cardiovascular system** |
| --- | --- | --- | --- | --- |
| 1 | Itch, flushing,  urticaria,  angioedema | - | - | - |
| 2 | Itch, flushing,  urticaria,  angioedema | Nausea, cramps | Rhinorrhea,  horseness,  dyspnoea | Tachycardia (rise ≥ 20bpm), hypotension (≥20 mmHg drop in SBP, Arrythmia) |
| 3 | Itch, flushing,  urticaria,  angioedema | Vomiting,  Defecation | Laryngeal oedema,  bronchospasm,  cyanosis | shock |
| 4 | Itch, flushing,  urticaria,  angioedema | Vomiting,  Defecation | Respiratory arrest | Circulatory arrest |

*bpm, beats per minute; mmHg,* millimetres of mercury; *SBP, Systolic Blood Pressure*
