# Supplementary material for: Desensitization in patients with hypersensitivity to platinum and taxane in gynecological cancers
Source: Cancer Med. 2023 Dec 22;13(1):e6840. doi: 10.1002/cam4.6840 (PMC10807606; doi:10.1002/cam4.6840)
Supplement: Supplementary file 5 — Table S2. [file CAM4-13-e6840-s004.docx]

**Table S2.** Comparison of the demographic and clinicopathologic characteristics between patients with taxane-related HSRs and no HSRs in EOC.

| Characteristics | All patients  (n=102)  n (%) | No HSR  (n=89)  n (%) | HSR to taxane  (n=13)  n (%) | P-value ^a^ |
| --- | --- | --- | --- | --- |
| Age at diagnosis (years) |  |  |  |  |
| Median | 64.1 | 66.6 | 57.0 | **0.036** |
| Range | 30.5-85.5 | 30.5-85.5 | 40.2-71.0 |  |
| FIGO stage |  |  |  |  |
| I+II | 23 (22.55) | 17 (19.1) | 6 (46.15) | 0.068 |
| III+IV | 79 (77.45) | 72 (80.9) | 7 (53.85) |  |
| Grade |  |  |  |  |
| 1 | 5 (4.9) | 5 (5.62) | 0 (0) | 0.126 |
| 2 | 2 (1.96) | 1 (1.12) | 1 (7.69) |  |
| 3 | 93 (91.2) | 82 (92.1) | 11 (84.6) |  |
| unknown | 2 (1.96) | 1 (1.12) | 1 (7.69) |  |
| Ethnicity |  |  |  |  |
| Caucasian | 91 (90.1) | 80 (89.9) | 11 (91.7) | 0.735 |
| Hispanic | 5 (4.95) | 4 (4.49) | 1 (8.33) |  |
| Asian | 5 (4.95) | 5 (5.62) | 0 (0) |  |
| Family history of gynecologic cancer | 38 (38.4) | 35 (40.2) | 3 (25) | 0.362 |
| BRCA status |  |  |  |  |
| BRCA 1 | 8 (7.84) | 7 (7.87) | 1 (7.69) | 0.484 |
| BRCA 2 | 4 (3.92) | 3 (3.37) | 1 (7.69) |  |
| No BRCA mutation | 55 (53.92) | 47 (52.77) | 8 (61.49) |  |
| No testing | 35 (34.3) | 32 (36) | 3 (23.1) |  |
| Type of operation |  |  |  |  |
| Primary debulking | 87 (87.9) | 10 (83.3) | 77 (88.5) | 0.566 |
| Interval debulking | 8 (8.08) | 1 (8.33) | 7 (8.05) |  |
| No operation | 4 (.04) | 1 (8.33) | 3 (3.45) |  |
| Residual disease |  |  |  |  |
| R0 | 63 (63) | 54 (62.1) | 9 (69.2) | 0.897 |
| R1 (0-10mm) | 29 (29) | 31 (27.4) | 3 (23.1) |  |
| R2 (>10mm) | 8 (8) | 7 (8.05) | 1 (7.69) |  |
| Concurrent platinum chemotherapy | 101 (99.0) | 88 (98.9) | 13 (100) | 1.000 |
| Lines of chemotherapy |  |  |  |  |
| Median | 1 | 2 | 1 | 0.683 |
| Range | 1-7 | 1-7 | 1-3 |  |
| Cycles of chemotherapy |  |  |  |  |
| Median | 6 | 6 | 5 | 0.055 |
| Range | 1-12 | 1-12 | 1-7 |  |
| Cumulative dose of platinum (mg) |  |  |  |  |
| Median | 1700 | 1700 | 1684 | 0.311 |
| Range | 1-4590 | 1-4590 | 280-2336 |  |

*^a^The p-values were calculated using Kruskal-Wallis test(medians) or Fisher's exact test(categorical data). A p-value <0.05 was considered significant.*

*EOC, epithelial ovarian, tubal, and peritoneal cancer; HSR, hypersensitivity reaction; n, number of patients; mg, milligram: mm, millimetre*
