# Supplementary material for: Desensitization in patients with hypersensitivity to platinum and taxane in gynecological cancers
Source: Cancer Med. 2023 Dec 22;13(1):e6840. doi: 10.1002/cam4.6840 (PMC10807606; doi:10.1002/cam4.6840)
Supplement: Supplementary file 6 — Table S3. [file CAM4-13-e6840-s001.docx]

**Table S3.** Comparison of the demographic and clinicopathologic characteristics between patients with platinum-related HSRs and no HSRs in EOC.

| Characteristics | All patients  (n=126)  n (%) | No HSR  (n=117)  n (%) | HSR to platinum  (n=9)  n (%) | P-value ^a^ |
| --- | --- | --- | --- | --- |
| Age at diagnosis (years) |  |  |  |  |
| Median | 67.1 | 67.2 | 56.6 | 0.246 |
| Range | 29.1-88 | 29.1-88 | 45.9-84.6 |  |
| FIGO stage |  |  |  |  |
| I+II | 29 (23) | 28 (23.9) | 1 (11.1) | 0.683 |
| III+IV | 97 (77) | 89 (76.1) | 8 (88.9) |  |
| Grade |  |  |  |  |
| 1 | 7 (5.56) | 7 (5.98) | 0 (0) | 1.000 |
| 2 | 3 (2.38) | 3 (2.56) | 0 (0) |  |
| 3 | 115 (91.3) | 106 (90.6) | 9 (100) |  |
| unknown | 1 (0.79) | 1 (0.85) | 0 (0) |  |
| Ethnicity |  |  |  |  |
| Caucasian | 116 (92.8) | 107 (92.2) | 9 (100) | 1.000 |
| Hispanic | 4 (3.20) | 4 (3.45) | 0 (0.00) |  |
| Asian | 5 (4.00) | 5 (4.31) | 0 (0.00) |  |
| Family history of gynecologic cancer | 48 (39.0) | 45 (39.5) | 3 (33.3) | 1.000 |
| BRCA status |  |  |  |  |
| BRCA 1 | 12 (9.52) | 10 (8.55) | 2 (22.2) | 0.374 |
| BRCA 2 | 7 (5.56) | 6 (5.13) | 1 (11.1) |  |
| No BRCA mutation | 60 (47.57) | 56 (47.82) | 4 (44.4) |  |
| No testing | 47 (37.3) | 45 (38.5) | 2 (22.2) |  |
| Type of operation |  |  |  |  |
| Primary debulking | 107 (86.3) | 7 (77.8) | 100 (87) | 0.449 |
| Interval debulking | 13 (10.5) | 2 (22.2) | 11 (9.57) |  |
| No operation | 4 (3.23) | 0 (0) | 4 (3.48) |  |
| Residual disease |  |  |  |  |
| R0 | 75 (61.5) | 71 (62.8) | 4 (44.4) | 0.273 |
| R1 (0-10mm) | 34 (27.9) | 31 (27.4) | 3 (33.3) |  |
| R2 (>10mm) | 13 (10.7) | 11 (9.73) | 2(2.22) |  |
| Concurrent taxane chemotherapy | 97 (83.6) | 88 (82.2) | 9 (100) | 0.352 |
| Lines of chemotherapy |  |  |  |  |
| Median | 1 | 1 | 5 | **<0.001** |
| Range | 1-7 | 1-7 | 1-7 |  |
| Cycles of chemotherapy |  |  |  |  |
| Median | 6 | 6 | 13 | **0.002** |
| Range | 1-24 | 1-24 | 5-20 |  |
| Cumulative dose of platinum (mg) |  |  |  |  |
| Median | 3760 | 3615 | 7550 | **0.019** |
| Range | 328-13470 | 328-13470 | 2070-11130 |  |

*^a^The p-values were calculated using Kruskal-Wallis test(medians) or Fisher's exact test(categorical data). A p-value <0.05 was considered significant.*

*EOC, epithelial ovarian, tubal, and peritoneal cancer; HSR, hypersensitivity reaction; n, number of patients; mg, milligram: mm, millimetre*
