# Supplementary material for: Desensitization in patients with hypersensitivity to platinum and taxane in gynecological cancers
Source: Cancer Med. 2023 Dec 22;13(1):e6840. doi: 10.1002/cam4.6840 (PMC10807606; doi:10.1002/cam4.6840)
Supplement: Supplementary file 7 — Data S1. [file CAM4-13-e6840-s005.docx]

**Supplementary material**

16-step protocol and alternatives [1, 2].

| Step | Solution | Rate ml/h | Time (min) |
| --- | --- | --- | --- |
| 1 | 1 | 1.25 | 15 |
| 2 | 1 | 2.5 | 15 |
| 3 | 1 | 5 | 15 |
| 4 | 1 | 10 | 15 |
| 5 | 2 | 2 | 15 |
| 6 | 2 | 5 | 15 |
| 7 | 2 | 10 | 15 |
| 8 | 2 | 20 | 15 |
| 9 | 3 | 5 | 15 |
| 10 | 3 | 10 | 15 |
| 11 | 3 | 20 | 15 |
| 12 | 3 | 40 | 15 |
| 13 | 4 | 10 | 15 |
| 14 | 4 | 20 | 15 |
| 15 | 4 | 40 | 15 |
| 16 | 4 | 75 | 15 |

Bag 1: 1/1000 Solution of full concentration

Bag 2: 1/100 Solution of full concentration

Bag 3: 1/10 Solution of full concentration

Bag 4: normal solution

For the 8 or 10 step accelerated protocol, desensitization is started at step 7 or 9, respectively.

**References**

1. Castells, M., C. Sancho-Serra Mdel, and M. Simarro, *Hypersensitivity to antineoplastic agents: mechanisms and treatment with rapid desensitization.* Cancer Immunol Immunother, 2012. **61**(9): p. 1575-84.

2. Villarreal-Gonzalez, R.V., et al., *Hypersensitivity reactions to anticancer chemotherapy and monoclonal antibodies: Safety and efficacy of desensitization.* J Oncol Pharm Pract, 2023: p. 10781552231189461.
