# Supplementary material for: Desensitization in patients with hypersensitivity to platinum and taxane in gynecological cancers
Source: Cancer Med. 2023 Dec 22;13(1):e6840. doi: 10.1002/cam4.6840 (PMC10807606; doi:10.1002/cam4.6840)
Supplement: Supplementary file 8 — Figure Captions. [file CAM4-13-e6840-s006.docx]

**Figure S1.** Desensitization procedure for immediate type hypersensitivity reactions to the chemotherapeutic agents taxane and platinum. *CTCAE=Common Terminology Criteria for Adverse Events; IDT=Intradermal test; pos=positive; neg=negative; AH=Antihistamines; CS=Corticosteroid*

**Figure S2.** Kaplan-Meier curves of (A) OS and (B) RFS of EOC patients with platinum-based chemotherapy and no hypersensitivity reaction (HSR) compared to patients with an HSR and successful desensitization and continuation of platinum chemotherapy. *OS=Overall survival; RFS=Recurrence-free survival; EOC= epithelial ovarian, tubal, and peritoneal cancer; HSR=hypersensitivity reaction.*

**Figure S3** Kaplan-Meier curves of (A) OS and (B) RFS of patients with taxane-based chemotherapy and no hypersensitivity reaction (HSR) compared to patients with an HSR and successful desensitization and continuation of taxane chemotherapy in EOC. *OS=Overall survival; RFS=Recurrence-free survival; EOC= epithelial ovarian, tubal, and peritoneal cancer; HSR=hypersensitivity reaction.*
